# AI driven pre-regulatory validation of PD-L1 analysis in lung cancer

**DOI:** 10.1038/s41598-025-28365-z

**Published:** 2025-11-28

**Authors:** Yasmine Makhlouf, Perry Maxwell, Paul O’Reilly, Alice Geaney, Jacqueline A. James, Manuel Salto-Tellez

**Affiliations:** 1 Precision Medicine Centre of Excellence, HealthSciencesBuilding, The Patrick G Johnston Centre for Cancer Research, Queen’s University Belfast, Belfast, BT9 7AE UK; 2 Sonrai Analytics, Concourse Building 2, Unit 2, Suite 1, Queens Road, Belfast, BT3 9DT UK; 3 Regional Molecular Diagnostic Service, BelfastHealthandSocialCareTrust, Belfast, BT9 7AE UK; 4 Integrated Pathology Unit, Institute of Cancer Research and Royal Marsden Hospital, London, SW7 3RP UK

**Keywords:** Cancer, Biomarkers, Computational biology and bioinformatics

## Abstract

The assessment of PD-L1 in lung cancer using the Tumour Proportion Score (TPS) is one of the cornerstones of immune-oncology, but it is open to inter- and intra-pathologist variation, particularly around the clinical thresholds of less than 1%*and* ⩾ 50%, which correspond to analytical thresholds less than 5% and between 40%-60%. In this paper we describe the development of a deep learn- ing (DL) tool to assist TPS calculation. To confirm ground truth values around the clinical thresholds, we used a validated multiplex immunfluorescence panel including PD-L1, CD68 and cytokeratin. Practically, the DL tool is designed to assist in highlighting cases about these thresholds around the 1% and 50% levels for manual review, and allowing a direct interpretation of inbetween scores. Us- ing such an assisted system, we highlight the potential use of such DL tools in providing a route to future clinical quantitation of tissue-based biomarkers.

## Introduction

The link of Programmed Death-1 (PD-1) and its ligand PD-L1 when expressed by malignant cells, results in the inhibition of T lymphocyte activation and pro- motes tumour growth (Reviewed by^1^). Antibodies blocking PD-1 or its ligand aim to restore innate immunity and hence are used for the treatment of various solid tumours^2,3^. Targeted PD-1 treatment and patient stratification is achieved by the reporting of PD-L1 tumour and/or tumour-associated immune cell expression, depending on tumour type, type of drug and scoring system.

PD-L1 Tumour Proportion Score (TPS) is derived from the number of positive viable tumour cells divided by the total number of viable tumour cells multiplied by 100 to express the result as a percentage. Clinical cut-offs for TPS have been established to indicate the likelihood of immunotherapy response in non-small cell lung carcinoma (NSCLC,^2^) and have been identified for first- and second- line treatments in both squamous cell carcinomas and adenocarcinomas (for full review see^4^). The current standard-of-care is the semi-quantitative scoring cal- culated by a pathologist. However, this is a difficult exercise prone to technical difficulties reviewed in^5^, which leads to a reported intra- and/or inter-observer (pathologist) variation. Indeed, the intra-and inter-observed variation reported is substantial (see^6–8^.

These studies also illustrate the fact that these discrepancies can be mitigated in part by training and coordination.

We have previously reported [see ref.^9^] that the diagnostic accuracy of the PD- L1 test can be improved using Digital Image Analysis (DIA), in this case machine learning using the open Source software QuPath^10^. In parallel, it has been shown that the use of multiplex immunofluorescence (mIF) coupled with quan- titative Image Analysis enables a better understanding of the tumour milieu in- trinsic to respective tumour samples^11,12^; importantly, a systematic review and meta-analysis of the literature suggested that mIF, in itself, holds a stronger clinical predictability than other test modalities, including IHC^13^.

In reporting, the use of DIA to improve diagnostic accuracy^5^ confirmed clusters of difficulty around the clinical thresholds of < 1% and between 40%- 60%.However, here we hypothesize that, artificial intelligence (AI) for the predic- tion of PD-L1 expression on a highly supervised deep learning (DL) model, may have an advantage as conceptually trained DL tools do not require case thresholds to be set. AI systems to assess PD-L1 have been shown to have a diagnostic ability

where^14^ developed an AI system using whole slide images (WSIs) to automat- ically assess TPS.

In a study carried out by^15^, a new AI-assisted scoring system for patholo- gists was tested for PD-L1 expression assessment in NSCLC. With the Aitrox AI segmentation model, PD-L1 expression was evaluated using TPS categorised into three levels. Aitrox results were comparable with the results of three of the five experienced pathologists, demonstrating the potential in assisting routine analysis of NSCLC by pathologists through scoring of PD-L1 expression.

In this work, we propose a new DL tool, developed as a result of a close collaboration between AI scientists and experienced pathologists at each step of the process^16^. Using this experience, we were able to help resolve problematic cell type identification of the ground truth values around the clinical threshold of < 1% using a validated mIF panel^17^ including PD-L1, CD68, a macrophage marker, and cytokeratin to highlight malignant epithelium in the multiplex panel. The DL tool is designed to score TPS from IHC images and provide scores for TPS intervals, highlighting clinically-relevant clusters around < 1% and 50% clinical cut-offs for manual assessment. In this way, DL would be an assistance to the pathologist in identifying difficult cases around clinical thresholds, known to be subject to variation in interpretation.

## Materials and methods

1100 anonymised digital images of PD-L1 stained NSCLC formalin fixed paraffin embedded (FFPE) tissue samples were acquired from the Northern Ire- land Biobank (NIB21-0066), along with 1100 anonymised digital images of the corresponding H&E. All images were linked to basic metadata which included the sample type, TPS status and the histological subtype. The cases included in this study were sourced from our routine histopathology diagnostic workflow, which infers that it reflects the spectrum of adenocarcinoma subtypes encountered in clinical practice. While specific details regarding the histologic subtype classi- fication of each adenocarcinoma case were not available, we have provided the total number of adenocarcinoma cases used. This cohort is therefore considered broadly representative of the histologic diversity observed in NSCLC adenocar- cinomas. In addition, the ground truth was provided by a group of specialist pathologists who have been working with this test since its early days^5^.

PD-L1 expression was assessed using the Ventana Roche SP263 assay and cases were representative of the four cellular pathology laboratories in the North- ern Ireland region.

**Fig. 1:**
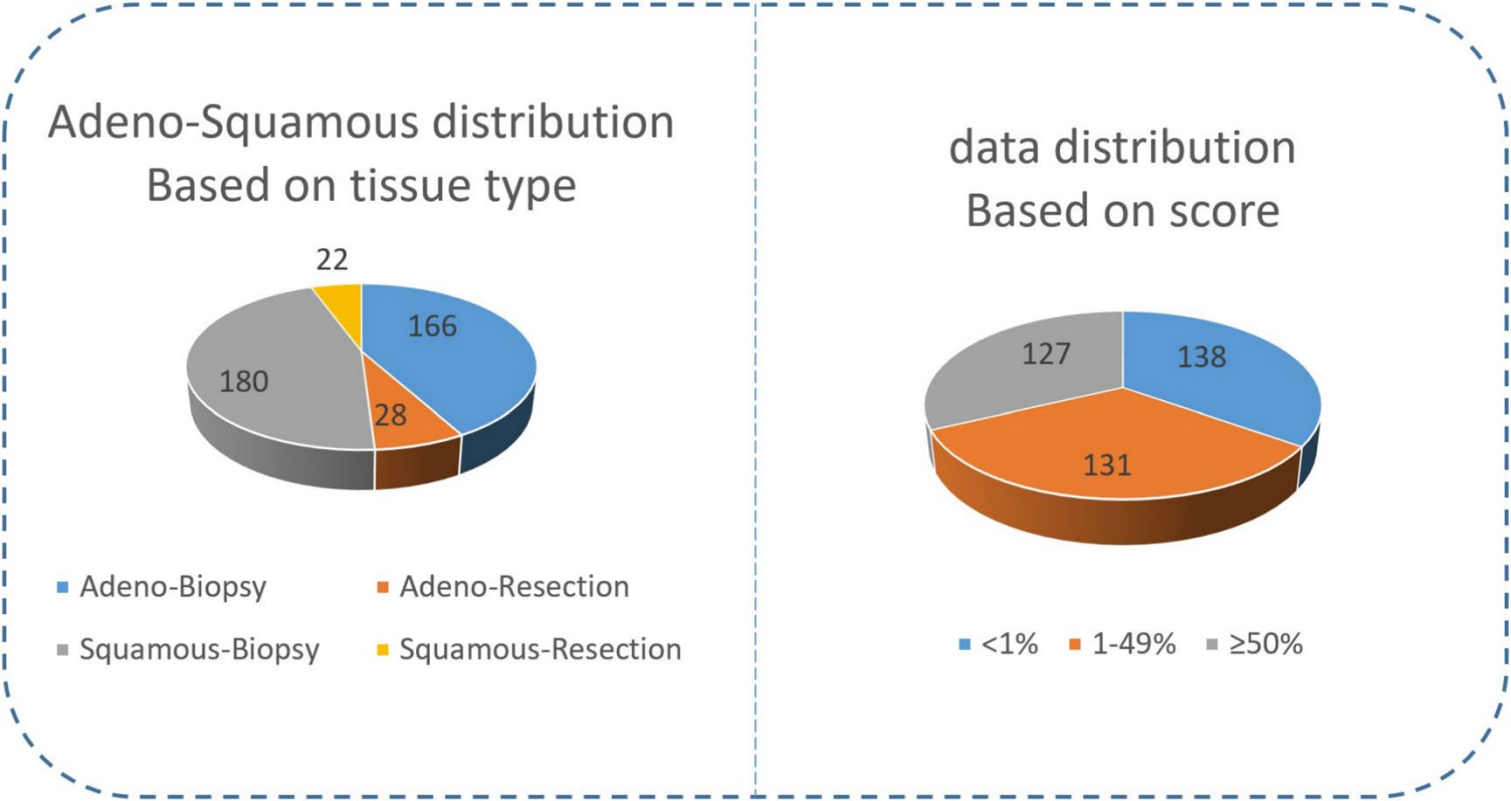
Data distribution. (**A**) Adenocarcinoma-Squamous (ADC-SCC) based distribution. (**B**) Ground Truth Score based distribution.

The images were used to TPS train, validate and test the AI model (see be- low), as well as the preliminary identification of tumour ROIs for the calculation of TPS. NSCLC (adenocarcinoma and squamous cell carcinoma) biopsy and re- section samples were used. All images were scanned using an Aperio AT2 scanner (scanner console ver- sion 102.0.7.5). Images were in svs format. Based on this file, QuPath version 0.3.2^10^ projects were organised by cancer type The Whole Slide Images (WSIs) were split into training, validation and testing sets. A group of 396 cases were retained for the development of the model and allocated in the following proportions across both biopsy and resection samples: 65% training, 16% validation, and 19% testing. Among these cases, 131 cases exhibited PD-L1 expression between 1–49%, and 127 cases showed expression ⩾ 50%. The positive cases were allocated as follows: 160 for training, 40 for val- idation, and 58 for testing. The choice of the split ratio was selected with respect to the size of the dataset. Random splitting of the dataset into training, valida- tion, and test sets is used as a common practice in machine learning, including image segmentation tasks to prevent bias in achieving randomness in the selec- tion of samples, and ensure a good generalisation. This number was sufficient to reach segmentation performance saturation, meaning that the accuracy of the segmentation stopped improving after reaching this number of patches, hence, no additional images were needed for the training process. Figure 1 illustrates the data distribution used based on Tissue-Type and Ground Truth TPS score.

Annotations were performed at two different levels:

- Cell level annotations: consisted of circling the PD-L1 positive intact tu- mour cells and PD-L1 negative intact tumour cells. Non-tumour PD-L1 positive and negative cells were identified and anno- tated as background within a pre-set box of 256 px × 256 px^18^.
- Regional (slide/case/patient) level annotations: for testing model perfor- mance in calculating TPS status. On a cohort of cases, all regions of tumour areas were identified and annotated as ROI(s).

Table 1 shows the data distribution through the different levels of annotations and consequently, the different levels of performance evaluation.

**Table 1:** Dataset distribution through the different performance evaluaition levels.

Purpose	Number of samples
Model devolopement-training and testing	396
Initial TPS test with precise TPS as GT	30
Patient level TPS test with interval TPS as GT + Multiplex integration	96

### Model development

A first version of the PD-L1 algorithm was trained based on three different classes: intact tumour negative; intact tumour positive; and background. Previous studies in our group determined the optimal deep learning tool for IHC analysis in general and described in detail in^16,19^, and^18^. These studies relied on intensive training and testing of different models and backbones from two distinct deep learning framework types used in segmentation tasks. We initially trained U-Net^20^, which is based on fully convolutional networks tailored for semantic segmentation, and Detectron2^21^, which belongs to the instance segmentation family built on region-based convolutional neural networks (R-CNNs). Each framework follows a different approach to object delineation, suited to specific segmentation tasks. In^19^ , UNet demonstrated superior performance in terms of sensitivity, averaging 65% compared to 59% for the Detectron model, when we consider Adam as the optimizer (Table 2). In^18^,the distinct training and evaluation processes of CD3, CD4, and CD8 T-cell biomarkers segmentation achieved an average sensitivity of 72.13% for FCN^22^, 65.23% for LinkNet^23^, 72.72% for DeepLabv3+^24^, and 81.12% for Resnet 35^20^. Based on these studies, we focused on UNet-based segmentation and further investigated its performance (see table 3) for PDL1, to finally adopt the UNet architecture described below. A more detailed tables and discussion of the overall evaluation process, including quantitative comparisons, are available in^16,18,19,25^.

**Table 2:** Summary of the best hyperparameter used to train the segmentation model.

Parameter	Value
Architecture	U-Net^20^
Backbones	ResNet-34^26^
Batch size	16
Normalization	0 − 255 to 0 − 1 OptimizerAdam
Learning rate	0*.*0001
Data augmentation	Rotation, Horizontal/vertical flipping Epochs 100
Loss function	Weighted cross entropy (WCE)

**Table 3:** Performance metrics of the proposed model.

Pixel level			Object level	
Accuracy	Sensitivity	Specificity	Precision	Recall
93.08	74.62	93.71	94.58	81.36

- Architecture : Unet
- Backbone : Resnet34
- Loss function : Weighted cross entropy loss (0.4, 0.9,0.9) (Background, Positive, Negative)
- Optimizer : Adam

A second version was the result of extensive training and testing of the adopted model from version 1, and relied on the additional dataset of a total of 396 cases (PD-L1 cells segmentation stage), from each case, four different patches (256 px x 256px) were generated, resulting in 1584 patches to train, validated and test the model at the PD-L1 segmentation stage. A U-Net architecture was used, consisting of an encoder and decoder block. The encoder block has eight layers, leveraging the ResNet-34 (He et al. (2016) pre-trained on ImageNet (Deng et al. (2009) to extract clinically relevant features like shape, texture, and intensity from patch images of T-cells. Residual blocks were employed to address the gradient vanishing problem during network training. The encoder utilized four Resnet intermediate layers, with the first layer using a 7 × 7 convolutional kernel to gen- erate 64 feature maps and the bottleneck layer producing 1024 feature maps with an 8 × 8 size (Makhlouf et al. (2024).

The decoder block consisted of eight decoding layers using Transpose convo- lutions. Its main purpose was to upsample the extracted feature maps to create binary segmentation masks for each T-cell’s biomarkers. Skip connections were employed, connecting the output of each encoder layer to the input of each de- coder layer, enabling the generation of precise cell segmentation boundaries. A threshold value of 0.5 was used to generate the masks.

We trained the model using the Adam optimizer with a learning rate of 0.0001 for 100 epochs and a mini-batch size of 8 and applied data augmentation such as rotation up to 30 degrees and horizontal/vertical flipping with a probability of 0.5 to introduce feature variability during training. To provide balance for pixels from the positive and negative classes (1,2), we applied the weighted cross- entropy (WCE) loss function by computing the weights of targeted cells and the background pixels. Table 2 summarizes the best hyperparameter used to train the segmentation model.

**Fig. 2:**
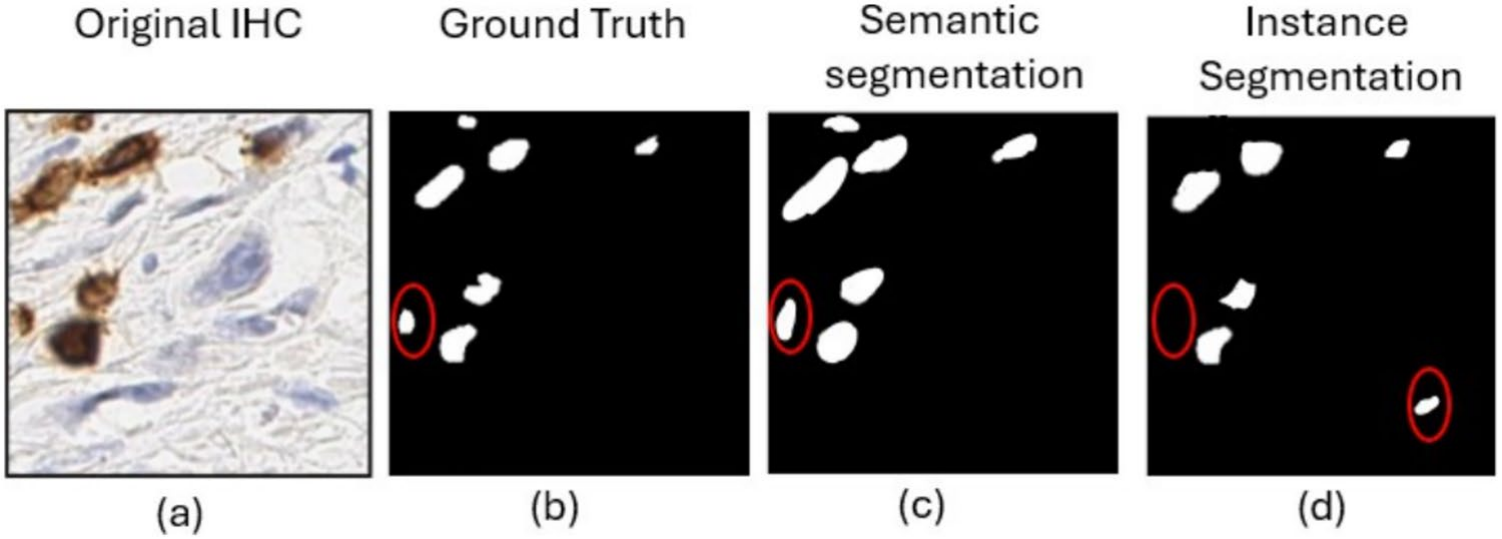
Example of CD3 biomarker cells segmentation (**a**) Original test images, (**b**) pathologist- annotated ground truth, (**c**) Semantic segmentation results (UNet), and (**d**) Instance Segmentation results.

**Fig. 3:**
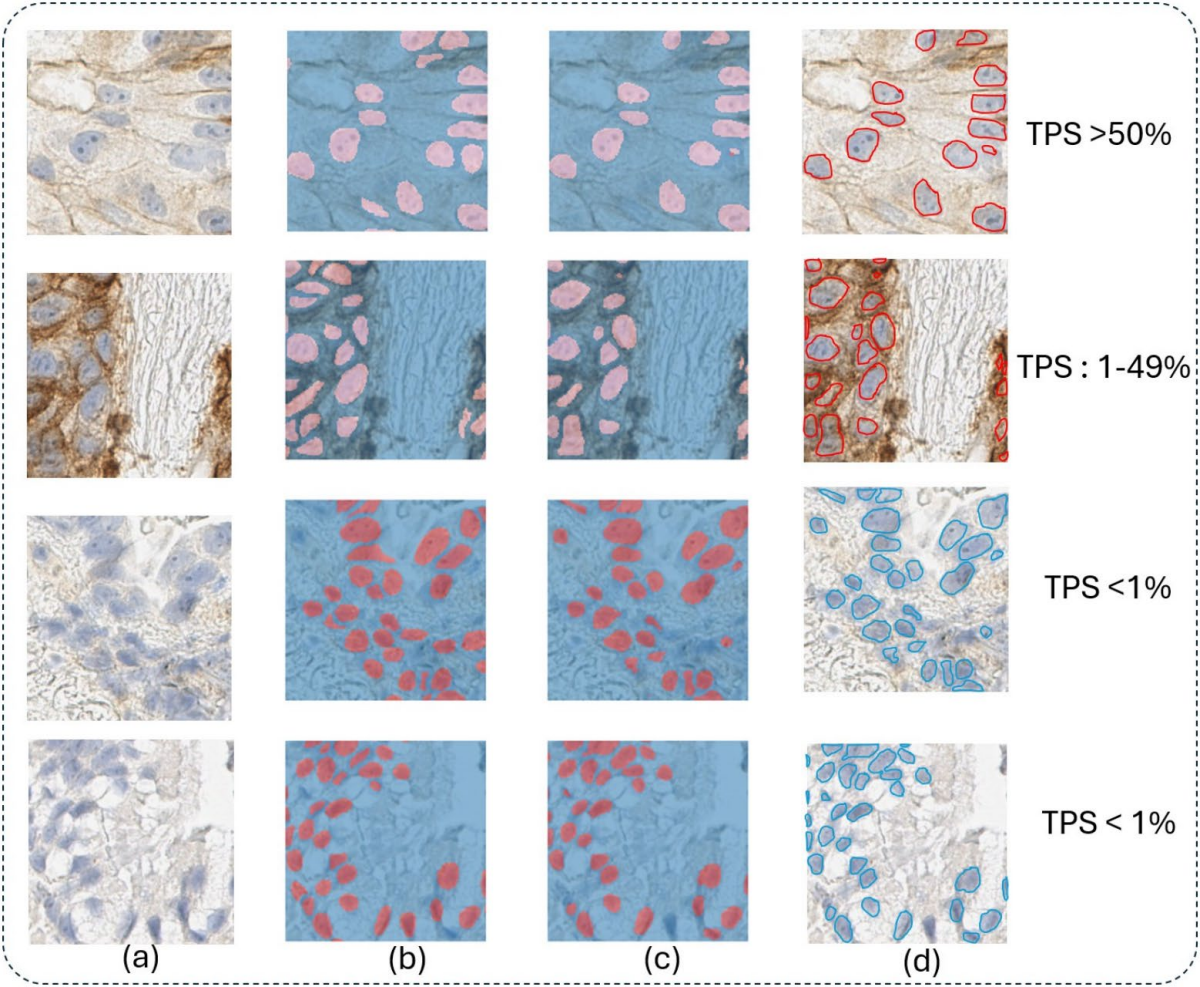
Representative PD-L1 IHC images illustrating (**a**).Original IHC images prior to the algorithm application, (**b**). Corresponding annotations overlayed on the same image, and (**c**). Final detections produced by the algorithm overlay overlayed on the same image, (**d**). Final detections highlighted as positive and negative PD-L1 tumor cells.

Trained on pathologist expert-annotated images where positive, negative and background are explicitly labelled, and through these series of downsampling (en- coder) and upsampling (decoder) layers, U-Net learns the hierarchical image fea- tures that distinguish morphological and textural patterns specific to the tumor cells, such as cell shape, nuclear morphology, and surrounding microenviron- ment, allowing it to separate cells from stroma. Once tumor cells are segmented, the model decides positivity based on staining intensity and distribution within the segmented tumor cell regions. Figures 2 and 3 include representative high- magnification images.

The segmented PD-L1 positive and negative cells are quantified separately us- ing a connected components method. This algorithm identified connected objects labeled as “1” for positive, and “2” for negative pixels belonging to each cell. A radius of four-pixel neighbors is considered for the connected components search. When applied to a selected ROI, the number of cells is estimated for every single patch generated through tiling the ROI and then summed up to calculate the TPS score as follows:

1 $${TPS\% = \frac{{{\mathrm{No}}.{\text{positive PD}} - {\mathrm{L1}} \ intact\ tumour\ cells}}{\ Total \ no \,of\,intact\ tumour\ cells} \times {1}00}$$

where *Total number of intact tumour cells* refers to the total number of PD-L1 intact tumour (positive and negative) cells in the ROI of WSIs. This estimation is evaluated for every patient.

## Performance evaluation and results

Model evaluation was assessed on three different levels:

- Pixel Level: each pixel from the ground-truth annotations was compared with each pixel from the corresponding model output image. We evaluated the pixel-level performance using three different metrics: accuracy, sensitivity, and specificity.
- Object level: performance metrics were computed by comparing ground truth an- notations and model output on a per-object basis; taking into account 4 pixel con- nectivity, each object from the ground-truth images (annotations) was compared with each object from the corresponding model output image. Precision and recall were used to evaluate how every two objects compare with each other.
- Patient-level: consisted of comparing the pathologist TPS with the algorithm es- timated TPS. Accuracy was the metric used to evaluate how these two estimates compare. Outcome from this phase of model development was based on data scien- tist and pathologist experience of the experiment metrics, and use of peer reviewed literature.

**Fig. 4:**
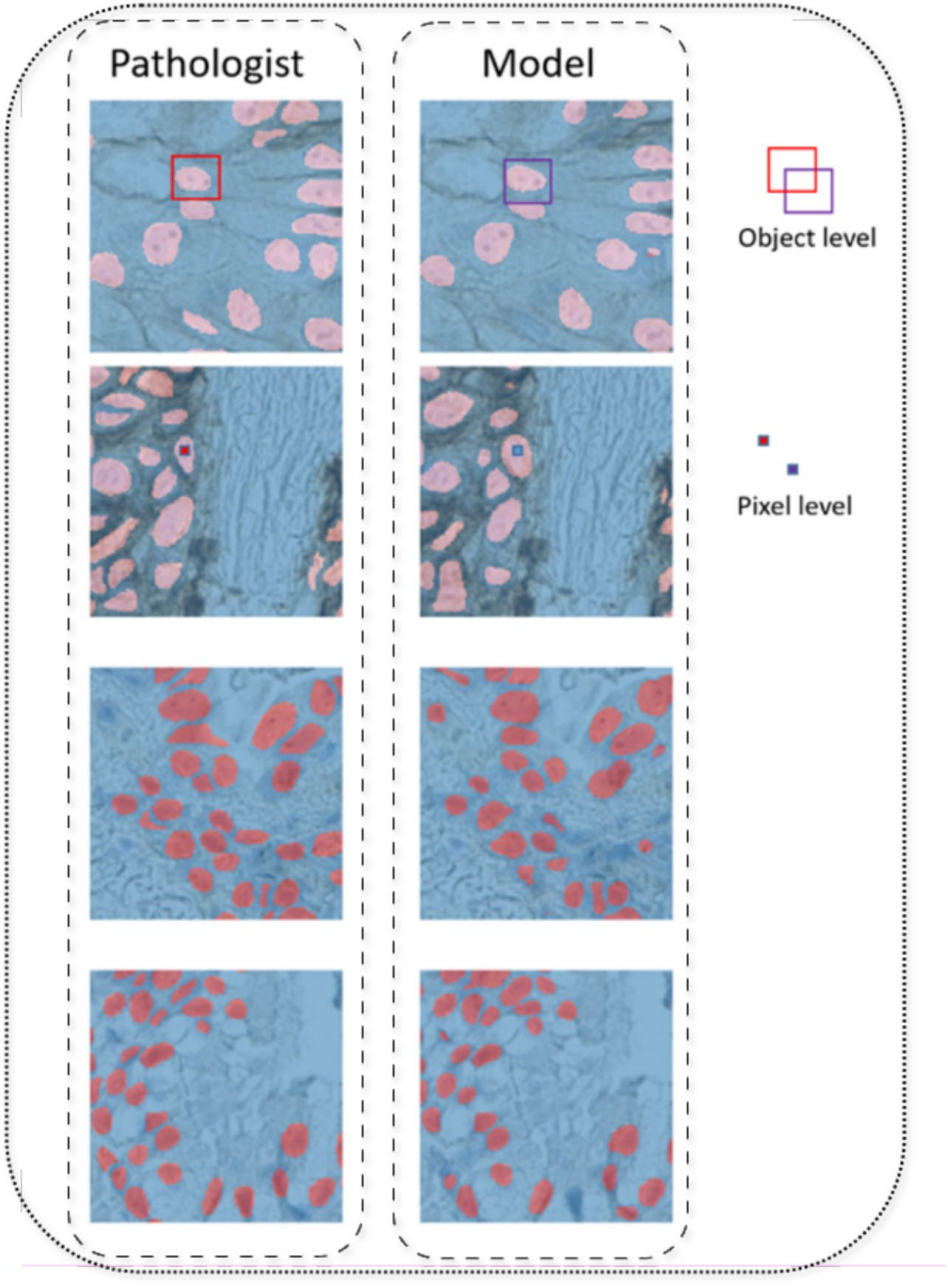
visual explanation of the difference between cell and object level performance evaluation.

Table 3 shows the Performance metrics of the proposed model at the pixel and object levels. Figure 3 illustrates the subcellular location considered as positive and negative expression,from three different overall TPS scores categories, with the original IHC images prior to algorithm application, the corresponding annotations overlayed on the same image, and the final detections produced by the algorithm. Figure 4 shows a visual explanation of the difference between cell and Object level performance evaluation.

**Fig. 5:**
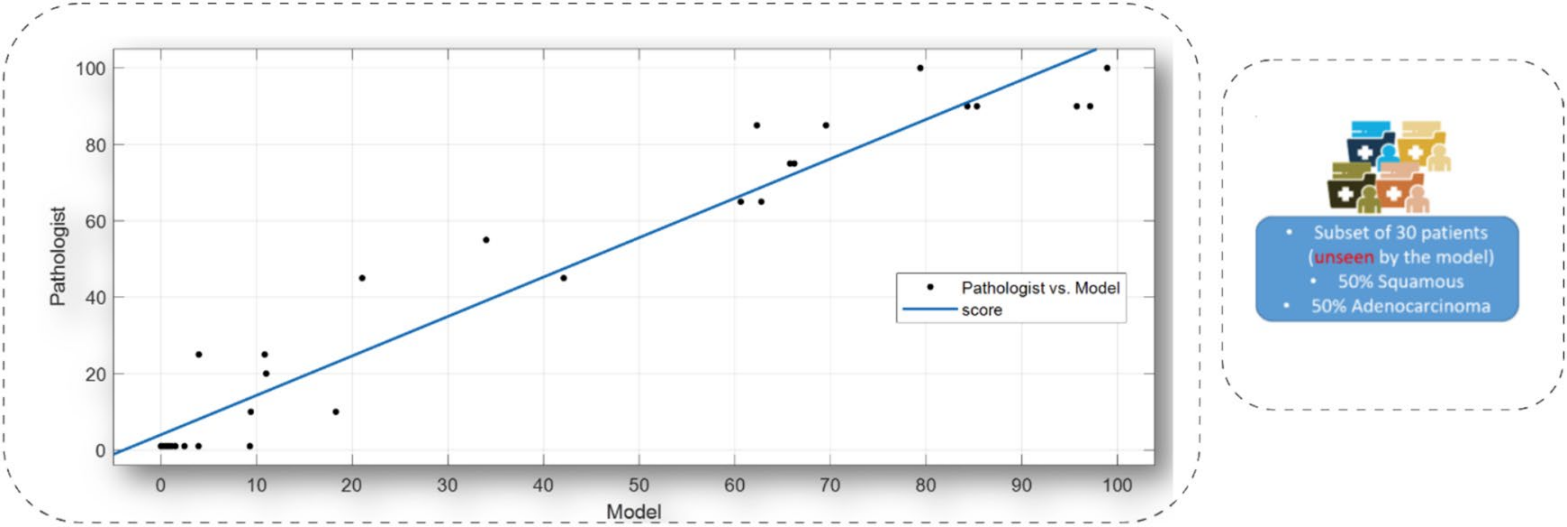
Pathologist TPS Vs Model TPS scores on a subset of 30 patients as an initial test.

**Fig. 6:**
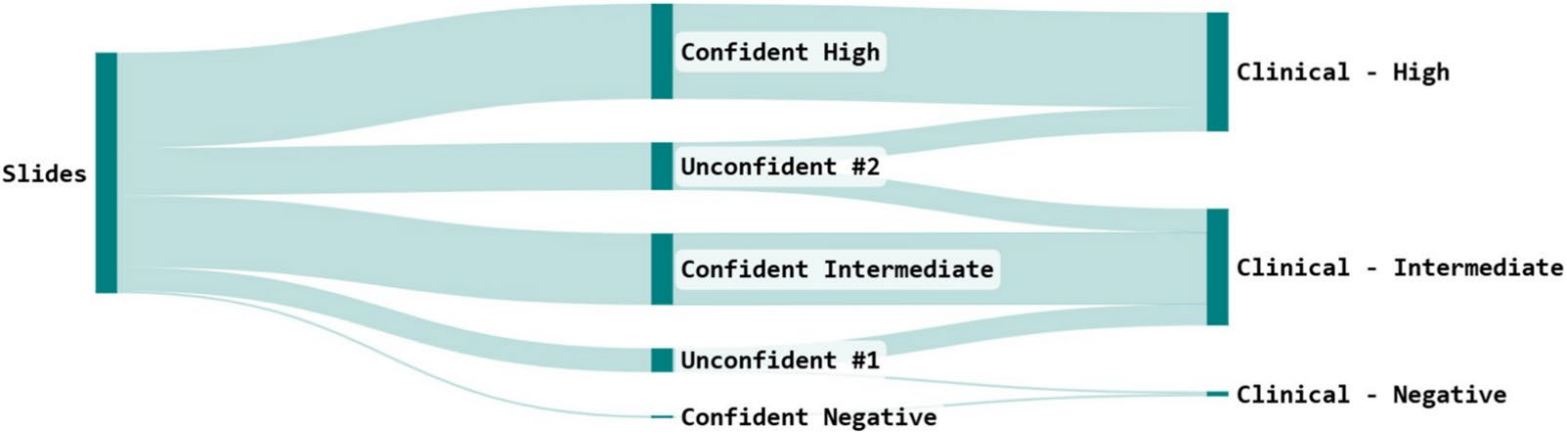
Sankey diagram showing the PD-L1 algorithm workflow defined with pathologists. The two ‘Unconfident’ categories require manual entry of the score by pathologists, with the confident categories subject to pathologist confirmation.

**Fig. 7:**
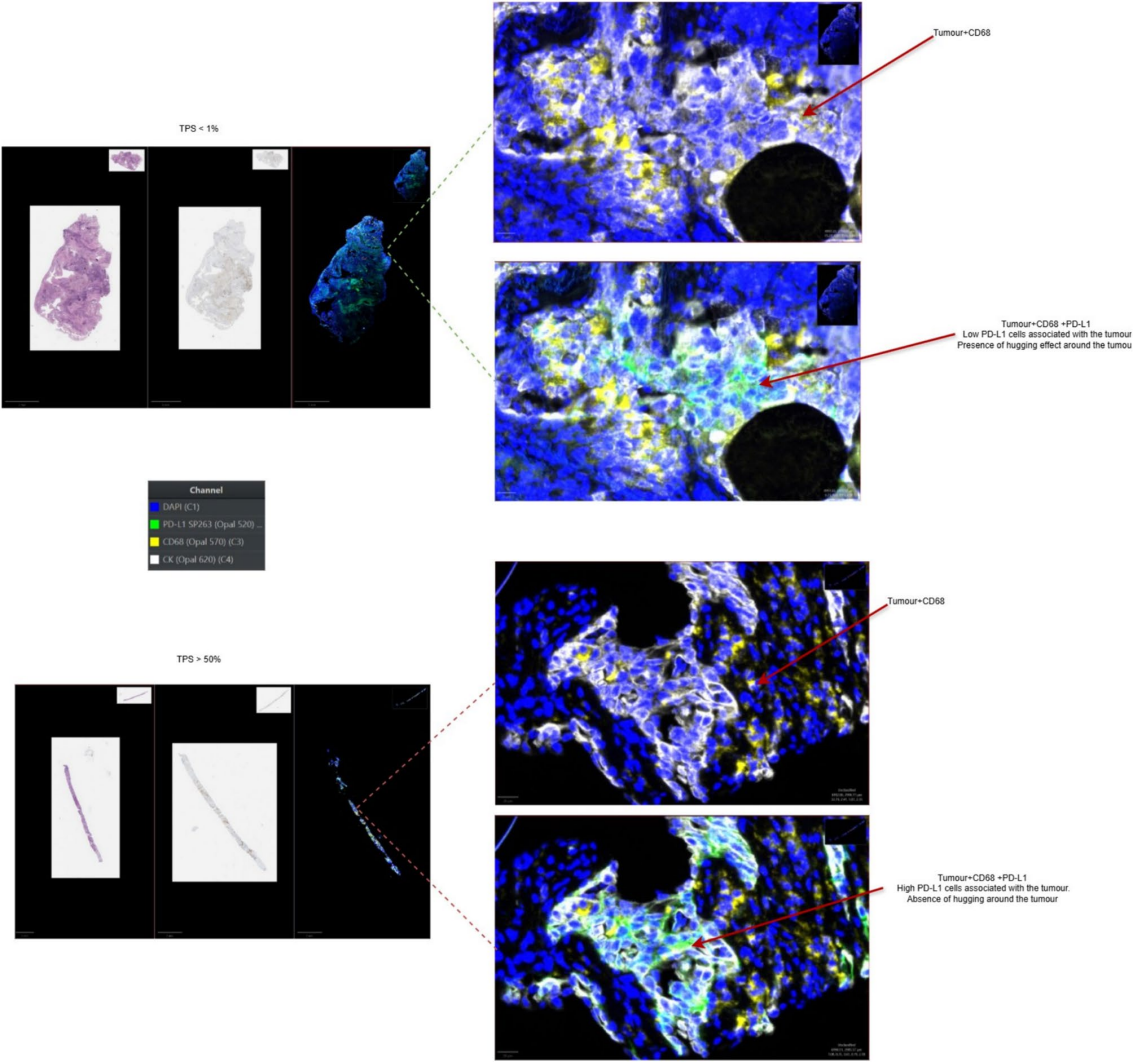
Multiplex merged images (PD-L1, CK, CD68) at high magnification, showing clear colocalization and spatial relationships between tumor cells and macrophages near the 1% and 50% clinical cut-offs.

**Fig. 8:**
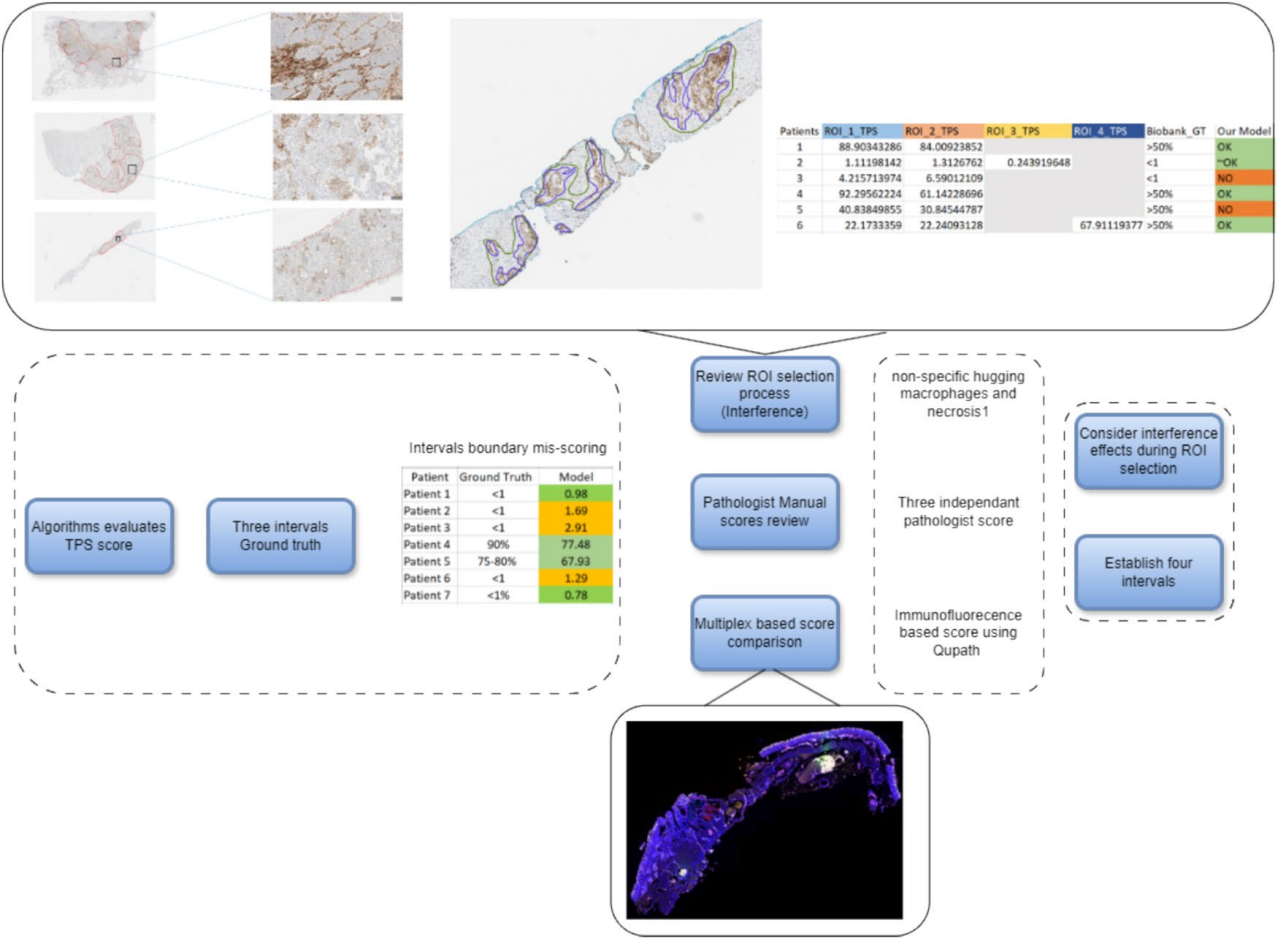
ROI review and new intervals establishment process.

**Fig. 9:**
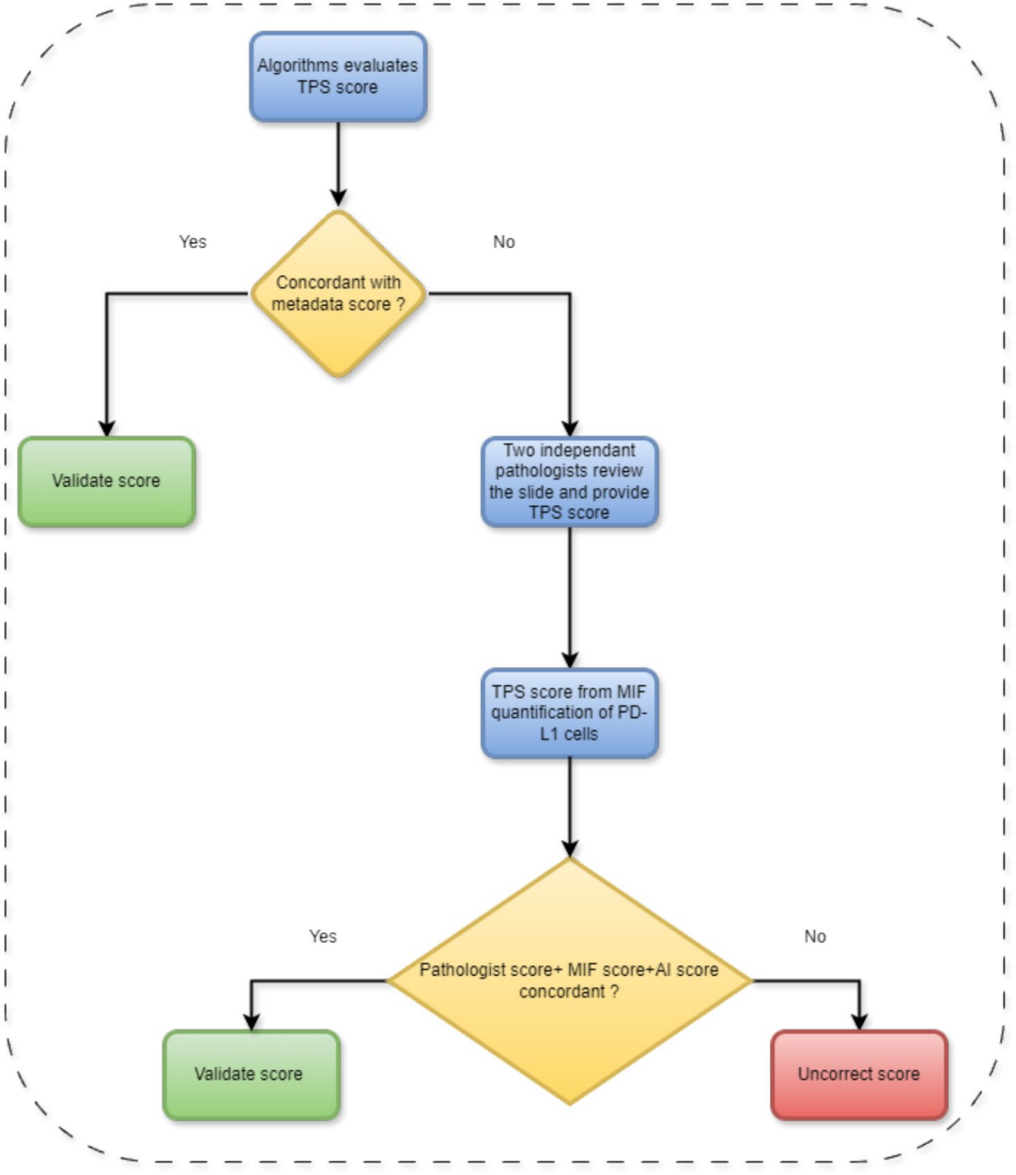
MIF integration principles to define ground truth TPS scores.

The quality of staining and scanning, along with the quality of annotations provided by the expert pathologists contributed to the performance of the model. An independent initial subset of 30 digital image samples, equally divided between adenocarcinomas and squamous cell carcinomas, where the pathologist assigned a TPS score as a percentage number to each case, considered these scores as our ground truth. The concordance re- sulted in a 96.97% correlation coefficient (see figure 5), which was an important positive indicator for evaluating the model on a larger cohort of images (Figure 6). The strong concordance between the pathologist annotations and the model segmentation output can be seen in figure 3 , showing the TPS as a result of the pathologist ground-truth annotations and corresponding mask predicted by the segmentation model (Figure 7). Our results support again the hypothesis that utilising this DL-based approach provides a robust segmentation and quantitation results (Figure 8). We looked at those cases around the < 1% clinical interval, using mIF to assess non- tumour and non-viable tumour PD-L1 expression on a sub-cohort of cases, the flowchart presented in figure 9 describes the mIF integration principles to define ground truth TPS scores. This confirmed the morphological assessment of non-tumour PD-L1 expression. Using CD68 as a macrophage marker in the panel and cytokeratin for tumour cells, the PD-L1 expression associated with non-tumour cells was highlighted. Figure 8 shows the hugging of PD-L1 around viable tumour clusters. Where such features can be difficult to distinguish on PD-L1 IHC alone, then we recommend that these would not be included in the annotation for algorithm PD-L1 assessment (Tables 4 and 5).

**Table 4:** Handover documents required under ISO13485 QMS and Design Control.

Document/artefact	Purpose/description
Design planning for model development	This aims to 1. Define the responsibilities and competencies of the staff involved in the model development 2. Identify the tools used in development, their purpose, and if any val- idation is required before the tool can be used 3. Define data protection controls for the data used in model develop- ment and ensure segregation of the training, validation and testing data sets, and protection of the model under development 4. Defines the tissue requirements and sample selection criteria, both from a clinical perspective (stage of disease, patient age, inclu- sion/exclusion of samples from vulnerable groups, tissue type, staining) but also from a data format (file type, image scanner, age of the sample) 5. Defines the statistical rationale for the training, validation and testing stratification 6. Quality control methods for samples and metadata, upon receipt and during annotation
Product definition	Defines the 1. Intended purpose of the model within the finished device 2. Target performance goals 3. Risks and control measures associated with the development that could have an impact on its overall performance, including bias in the data set, overfitting, underfitting etc 4. Technical limitations of the model that need to be considered for the finished product—limit of analysis for minimum pixels or equivalent
Annotation procedure	Includes the 1. Scientific rationale for the annotation to be used for model training 2. A step by step guide to perform the annotation 3. Definition of the acceptance criteria for an annotated sample, and how issues with samples should be handled so they are excluded from training 4. Definition of how records will be maintained during the annotation process (relating to the annotation or sample information that may be relevant during model training and error analysis) All staff performing the annotation should meet the minimum com- petency requirements defined in the planning stage, and have training records for the annotation procedure to confirm competency
Data pre-processing procedure	Defines the technical preprocessing used for the algorithm (e.g. patch sizes in pixels, channel format, normalization etc.), allowing the technical integration of the algorithm into the deployment platform As there is a chance that data may be lost during the annotation stage, and during the pre-processing stage, the pre-processing procedure should describe how new data can be added after initial pre-processing has been performed The procedure should describe how the correctness of the interim steps and the final results are assessed through risk-based evaluations. Where outliers are identified, they should be investigated
Machine learning operations	Machine Learning Operations This document defines the model training pipeline and can refer out to the annotation and pre-processing procedures where they are separate, but should include at a minimum labelling operations, tile extraction, compute of labelled features, where thresholds are present like this subject model, a method to train those should be defined, and model hyperparameters to be used in training, datasets and thresholds defined Control of the model is critically important, so version control mecha- nisms must be defined, alongside how the model will be published Requirements for error analysis must be included in the ML operations to ensure that where the model result does not align with the GT, suitable analysis is performed to understand the difference and drive retraining, wider thresholds for uncertain boundaries or identification of additional risk control measures in the finished product (such as limiting the in- tended use of the clinical product (to exclude biopsies for e.g.), use related software controls, or provision of information for user training and the instructions for use)
Model specification report	A summary of the model development activities, with details of the final model version, definition of the final data used to train, validate and test the model, model parameters, performance metrics (such as sensitivity, specificity, test replacement rate), summary of error analysis activities and the results/decisions made as a result, details of how change con- trol will be managed for future versions and any details about the model that are relevant to it working within a finished device (such as con- firmed limit of analysis, risks which need software driven risk controls, cybersecurity, details of remaining anomalies)

**Table 5:** Performance of previous studies considering three TPS intervals.

Methods	< 1	1–49	TPS Intervals
			> 50
^32^	65		92.60
^15^	85.29	77.97	72.73
^28^	64.80		94.40

A review of the original pathologists’ scores led to establishing four new TPS score intervals, recommending that with algorithm TPS scores falling 1% − 5% and 40% and 59.9%, should be reviewed by the reporting pathologist(s). All others would be acceptable. Table 6 presents the performance of our method, considering three TPS intervals, and four TPS intervals. A review of the Region of Interest (ROI) selection for algorithm assessment, helped provide pathologists with a better insight on the effect of including non-specific protein “hugging”, as well as PD-L1 protein in macrophages and necrosis 8. The process of validation is detailed in 9.

**Table 6:** Performance of our proposed method, considering three TPS intervals, and four different intervals.

Methods	Three int			Four int			
TPS score%	< 1	1–49	> 50	< 4.9	5 − 39.99	40 − 59.99	> 60
	47.16	95	95	88.3	90.90	66.67	71.43

### Translating the AI algorithm into the clinic

Development of a robust, performant algorithm in the research lab is only the first step towards the deployment of that algorithm in the clinic. As detailed by Geaney et al^27^, there are many regulatory and other milestones which must be achieved before an algorithm may be used safely on a particular patient population, with the core algorithm being only one piece of the jigsaw. This section describes work done to translate this PD-L1 algorithm.

### Algorithm handover

The PD-L1 algorithm detailed in sections 3 and 4 was developed for transfer into a clinical product (Sonrai Diagnostics PDL1), developed under design control within an ISO 13485-certified Quality Management System (QMS). The handover of the algorithm required the following artefacts, documentation and information, reviewed and approved within the QMS, desribed in table 4.

### Workflow

The algorithm developed, once it reaches the required performance detailed above, needs to be implemented within a workflow which will add value to pathologists scoring PD-L1 in the clinical context. This takes into account the context of the distinct clinical categories reported for a case < 1%, 1 *−* 49%, ⩾ 50%, and allows for manual refinement of scores around these critical thresholds. The engineering team worked with the pathol- ogists in the PMC to define the workflow in such a way that pathologists must check and manually score marginal cases, as a risk control measure against any algorithm inaccuracies. This workflow is shown in figure 6.

Regulatory requirements for medical devices mandates for a clear definition of the intended purpose of the device at the beginning of development, and this should be taken into account regarding what is accepted as state of the art and thus drive downstream design decisions. The Sonrai Diagnostics PDL1 device is intended to be an assistive device, and keeps the Human-in-the-Loop.

The interface to enable this workflow was developed using formative UX studies in- volving pathologists and UX designers - resulting in a simple and intuitive UI for an- notating the ROI, running the algorithms, viewing the results and reporting on the case. Arising from this work two particular features of the product were developed to augment the algorithm.

Firstly, a facility to upload the H&E slide image for the case and view it alongside the IHC slide to assist in drawing the ROI was added. This was added in response to the identification of a risk associated with close proximity macrophages during a study of interfering factors carried out by PMC during their internal validation of results for handover.

Secondly, as an assist to the pathologist a result overlay indicating the positivity of individual tumour cells, as determined by the algorithm was developed. This involved post-processing the results of the algorithm described here to determine the centroid of the detection, and displaying as an overlay to the IHC image on the results.

The GUI design and workflow enable the clinical user to review the outputs of the algorithm with the overlay to make a decision to agree or disagree with the algorithm result. Depending on the clinical user’s decision of agree or disagree, they are provided with a number of options to process close the case or take further action with the Sonrai Diagnostics system (e.g. reannotate or get a second opinion), or route the sample for the current gold standard assessment via manual scoring.

### Cybersecurity

As discussed in table 4 above, appropriate data protection and segregation of the train- ing, validation and test data is critical to ensure that data poisoning, evasion, data breach or model extraction does not occur. An algorithm without appropriate cybersecurity controls in place is vulnerable to ad- versarial attack; these attacks aim to change the classification or decision of the algorithm, or alter the returned supporting decision information that is intended for the clinical user to interpret the algorithm result - for Sonrai Diagnostics PDL1 this is the coordinates of the overlay. Researching publicly available sources for known adversarial threats enables a more thorough security risk assessment to be developed. The security risk assessment will identify a hazard that if realised could result in a patient safety risk and as such should be treated as a risk under EN ISO 14971 and IVDR and reduced as far as possible. The security and safety risk assessment aims to identify device and environmental controls which can protect the algorithm when it is in clinical use. The cloud based nature of Son- rai Diagnostics aims to reduce the potential for risk to a hospital labs network, and thus reducing the risk to other devices and data in that network.

## Discussion and conclusion

In this study, we present a Deep Learning (DL) model for the evaluation of the Tu- mour Proportion Score (TPS) in non-small cell lung carcinoma (NSCLC), both adenocar- cinoma and squamous cell carcinoma. Through the close collaboration of pathologist and computer scientist, we have shown that a highly supervised, trained model provided with expertly-selected PD-L1 positive and negative objects (cells), and TPS values, leads to a useful tool in assisting in the PD-L1 evaluation of NSCLC.

We relied on muli-version models development, to set the optimal deep learing archi- tecture and parameters^16,18,19^ to segment PD-L1 positive and negative cells . Followed by extensive training and testing of the optimal model, using hand generated annotations by experienced pathologists. The segmented PD-L1 positive and negative cells are quantified separately. When applied to a selected ROI, the number of cells is estimated for every single patch generated through tiling the ROI and then summed up to calculate the TPS score (see equation (1)).

The TPS output values on the clusters of difficult decision-making regarding the PD- L1 status around the 1% and 50% values recognised by^5,28^, and^15^, are recom- mended using this tool to be manually confirmed by the reporting pathologist, and if necessary to be re-evaluated and new value reported. This epitomises human-oversight when using such tools in clinical decision-making^29^. Recent studies have confirmed this challenge, as reported by^30^, interobserver agreement dropping to moderate at the <1% threshold (*k*=0.52), despite high concordance at ⩾ 50% (*k* = 0.87). Similarly,^31^ demonstrated that while AI-based TPS correlated strongly with pathologists (p=0.93), concordance was only 52% for TPS < 1% versus 85–89% for higher categories. Our use of mIF to assess non-tumour and non-viable tumour PD-L1 expression confirmed through spatial differential expression of PD-L1 cell type expression, the ground truth for the evaluation of both PD-L1 and the performance metrics of the DL model in their assessment. We found that in areas of macrophage expression and protein close prox- imity of PD-L1 the decision to ignore the non-tumour or non-tumour viability by the pathologist was correct, and that the given ground truth value of < 1% was correct. The mimicking of this decision by the model further confirmed the value of using patholo- gist expertise in this highly supervised manner for sample selection in model training and development. Figure 7 illustrates representative images from this panel, including Multi- plex merged images (PD-L1, CK, CD68) at high magnification in Qupath (^10^), showing clear colocalization and spatial relationships between tumor cells and macrophages. It provides a visual demonstration of how mIF supports the pathologist in establishing the ground truth annotation, particularly in complex cases with TPS around 1% and 50%, where macrophage PD-L1 expression may confound tumor cell scoring. The multiplex immunofluorescence (mIF) images served as visualization and validation tools for the pathologists. Specifically, by activating the CD68 and PD-L1 channels, pathologists were able to carefully examine the close proximity of macrophages expressing PD-L1 around tumor cells. This information was used to validate and refine the ground truth annotations for PD-L1 positivity in tumor cells, ensuring that macrophage PD-L1 expression was not mistakenly attributed to tumor cells.

Our model was trained on PD-L1 NSCLC FFPE tissue samples stained with PD-L1 using the SP-263 (Roche) system, and is designed to work following tumour annotation by a pathologist with a correlation coefficient of 96.97% on 30 WSIs. As a comparison,^32^ developed an AI system using WSIs of the 22c3 assay to automatically assess TPS of PD-L1 expression based on a DL model of tumor detection. The model was further trained on 100 WSIs of SP263 IHC staining and compared with the TPS results of con- sensus from specialist pathologists. In addition, the TPS-AI and TPS-pathologists were compared using cutoffs at 1 and 50%. The results showed moderate and excellent agree- ment of approximately 0.65 at *TPS* < 50% and 0.926 at *TPS* < 50%. red^31^ evaluated an AI-powered TPS analyzer on 802 NSCLC slides and observed strong overall concor- dance with pathologist scores (Spearman *p* = 0.925), with individual cut-off concordances of 52.4% (< 1%), 89.3% (1–49%), and 85.7% (< 50%)^15^ considered that the PD-L1 TPS to be clustered into three categories: nega- tive (*TPS* < 1%), low (*TPS*1–49%), and high (*TPS* < 50%), and the AI model showed 85.29%, 77.97% and 72.73% for the three categories, respectively^28^ verified the treatment-decisive concordance of automated PD-L1 scores with that of human investigators, they compared PD-L1 scores using typical cutoffs described in the literature. As cutoffs, they used *TPS* ⩾ 1% and *TPS* ⩾ 50%. Human–machine concor- dance was best for *TPS* < 50% with 94.4%. Table 5 summarizes the results considering the intervals proposed by the authors.

Overall, the PD-L1 tool is designed to work with the pathologist, depending on expert ROI annotation, followed by running of the algorithm. It is intended that those results around 1% and 50% should be reviewed by the pathologist, confirm or refuted with mod- ified evaluations reported. PD-L1 TPS values outside of these would be accepted unless otherwise overridden by the pathologist. This will lead to a significant reduction in work- load and in inter- and intra- reviewer variation. This assist to pathology of reporting of PD-L1 appears to be a favoured means of machine learning integration.

## Data Availability

The dataset generated and/or analysed during the current study are not publicly avail- able, and was provided by the Northern Ireland Biobank under *NIB* 19 */* 310, which relies on an ethical framework for collection and access to tissue samples. Data availability is subject to an application to the Northern Ireland Biobank, upon reasonable request from the authors.
